# Histone deacetylase-3 interacts with ataxin-7 and is altered in a spinocerebellar ataxia type 7 mouse model

**DOI:** 10.1186/1750-1326-8-42

**Published:** 2013-10-27

**Authors:** Carlotta E Duncan, Mahru C An, Theodora Papanikolaou, Caitlin Rugani, Cathy Vitelli, Lisa M Ellerby

**Affiliations:** 1 Buck Institute for Research on Aging, 8001 Redwood Blvd, Novato, CA 94945, USA

**Keywords:** HDAC, Ataxin-7, Spinocerebellar ataxia type 7, Polyglutamine

## Abstract

Spinocerebellar ataxia type 7 (SCA7) is caused by a toxic polyglutamine (polyQ) expansion in the N-terminus of the protein ataxin-7. Ataxin-7 has a known function in the histone acetylase complex, Spt/Ada/Gcn5 acetylase (STAGA) chromatin-remodeling complex. We hypothesized that some histone deacetylase (HDAC) family members would impact the posttranslational modification of normal and expanded ataxin-7 and possibly modulate ataxin-7 function or neurotoxicity associated with the polyQ expansion. Interestingly, when we coexpressed each HDAC family member in the presence of ataxin-7 we found that HDAC3 increased the posttranslational modification of normal and expanded ataxin-7. Specifically, HDAC3 stabilized ataxin-7 and increased modification of the protein. Further, HDAC3 physically interacts with ataxin-7. The physical interaction of HDAC3 with normal and polyQ-expanded ataxin-7 affects the toxicity in a polyQ-dependent manner. We detect robust HDAC3 expression in neurons and glia in the cerebellum and an increase in the levels of HDAC3 in SCA7 mice. Consistent with this we found altered lysine acetylation levels and deacetylase activity in the brains of SCA7 transgenic mice. This study implicates HDAC3 and ataxin-7 interaction as a target for therapeutic intervention in SCA7, adding to a growing list of neurodegenerative diseases that may be treated by HDAC inhibitors.

## Introduction

Spinocerebellar ataxia type 7 (SCA7) is a rare neurodegenerative disease caused by a trinucleotide repeat expansion in the ataxin-7 gene [1-3]. SCA7 is one of nine known polyglutamine (polyQ) diseases characterized by aggregation of polyQ-expanded protein, nuclear inclusions and repeat-dependent neuronal toxicity [4,5].

Ataxin-7 protein is ubiquitously expressed in the brain with cytoplasmic and nuclear staining [6,7]. Ataxin-7 functions as part of two known transcriptional repressor complexes: the Spt/Ada/Gcn5 acetylase (STAGA) chromatin remodeling complex with histone acetyltransferase (HAT) activity [8]; and the TATA-BP free TAF-containing complex (TFTC) [9,10]. In SCA7 patients there is increased localization of ataxin-7 to discrete nuclear inclusions [6,7,11,12]. Aggregation of polyglutamine-expanded ataxin-7 into the nucleus, concomitant with neurodegeneration and transcriptional deregulation, is also a feature of SCA7 transgenic mouse models [13-16].

Transcriptional deregulation in polyglutamine diseases has been associated with acetylation state changes, by alteration of the HAT activity of member complexes [17] or by direct interaction of ataxins with histone deacetylases (HDACs), in particular HDAC3 [18,19]. HDAC3 is an integral constituent of multiple co-repressor complexes that activate HDAC3 and are recruited by transcription factors to alter histone acetylation and repress transcription [20-22]. HDAC3 shuttles between the cytoplasm and nucleus [23], altering activity of transcriptional repressor complex target genes [24]. HDAC3 is highly expressed in the brain [25] and is essential for viability [26]. In cell studies, high levels of HDAC3 are neurotoxic whereas HDAC3 suppression protects against neuronal cell death [27]. Further, this appears to be a modular switch in which HDAC3 interaction with HDAC1 promotes survival or death [28]. This is consistent with the proposed neuroprotective nature of HDAC inhibitors, including those targeting HDAC3, in reducing pathological behavioral phenotypes and neurodegeneration in mouse models [29-32].

Previous work by our lab and others has shown that ataxin-7 posttranslational modification (PTM) increases ataxin-7 stability, effecting aggregation and toxicity of the polyQ-expanded protein [16,33], with deacetylation influencing stability of ataxin-7 although the enzyme that was responsible for this effect was not determined. In this study we found that HDAC3 enhanced PTMs and the stability of ataxin-7, with evidence that ataxin-7 and HDAC3 physically interact and colocalize. Their association enhances cellular toxicity in a polyQ-dependent manner. HDAC3 displays robust expression in neurons and glia in the mouse cerebellum. We detect increased levels of HDAC3 in SCA7 mice as well as altered lysine acetylation levels and deacetylase activity in the brains of SCA7 transgenic mice, indicating that the association between HDAC3 and ataxin-7 may be a pathological feature of the disease.

## Results

### HDAC3 effects ataxin-7 in a SCA7 cellular model

**Figure 1:**
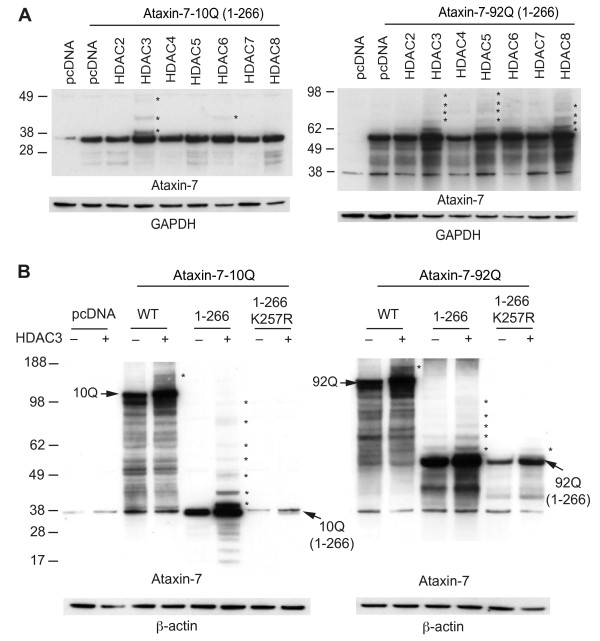
**HDAC3 stabilizes ataxin-7 and enhances post-translational modifications. (A)** Ataxin-7-10Q (1–266) and ataxin-7-92Q (1–266) were transiently transfected with HDAC constructs [2-8] in HEK293T cells for 72 hrs. Lysates were collected and separated via SDS-PAGE on 4-12% bis-tris gels. Western blot transfer and analysis with an antibody to ataxin-7 (PA1-749) revealed the presence of bands of expected molecular weight for the ataxin-7 stop constructs as well as bands of higher molecular weight indicated by asterisks, particularly evident in the HDAC3 co-tranfection. GAPDH was used as a loading control. **(B)** Ataxin-7-10Q, ataxin-7-10Q (1–266), ataxin-7-10Q (1–266) K257R, ataxin-7-92Q, ataxin-7-92Q (1–266) or ataxin-7-92Q (1–266) K257R were co-transfected with vector control or HDAC3 in HEK293T cells. Western blot analysis of cellular lysates with ataxin-7 (PA1-749) antibody revealed the presence of bands corresponding to the wild type, expanded and ataxin-7 1–266 stop mutant. Bands of higher molecular weight were dominant when ataxin-7 was co-transfected with HDAC3 (indicated by asterisks). Note that these bands are diminished in the SUMOylation-deficient ataxin-7 mutant (K257R). β-actin was used as a loading control.

The effect of HDACs on ataxin-7 stability and post-translational modifications was examined using a panel of HDAC constructs co-expressed with the N-terminal fragment of ataxin-7 (wild type and expanded) in HEK293T cells [16]. HDAC3 co-expression had the most obvious effect on ataxin-7 – upregulating the levels of ataxin-7 as well as dramatically increasing the number of high molecular weight bands immunoreactive to the ataxin-7 antibody (Figure 1A). Other HDACs had much more subtle effect on ataxin-7 stability and post-translational modification and so we chose to focus on HDAC3.

The effect of HDAC3 on ataxin-7 protein levels was examined further by western blotting of the full-length ataxin-7, N-terminal fragments and a mutant resistant to modification (K257R) (Figure 1B). HDAC3 co-expression with both wild type ataxin-7-10Q and polyQ-expanded ataxin-7-92Q lead to an increase in high-molecular weight bands of ataxin-7 as well as a marked increase in protein expression. While HDAC3 did increase the stability of ataxin-7 K257R, post-translational modifications were diminished, indicating that HDAC3 co-expression targets the SUMOylation and/or acetylation site, K257.

### Ataxin-7 physically interacts with HDAC3

**Figure 2:**
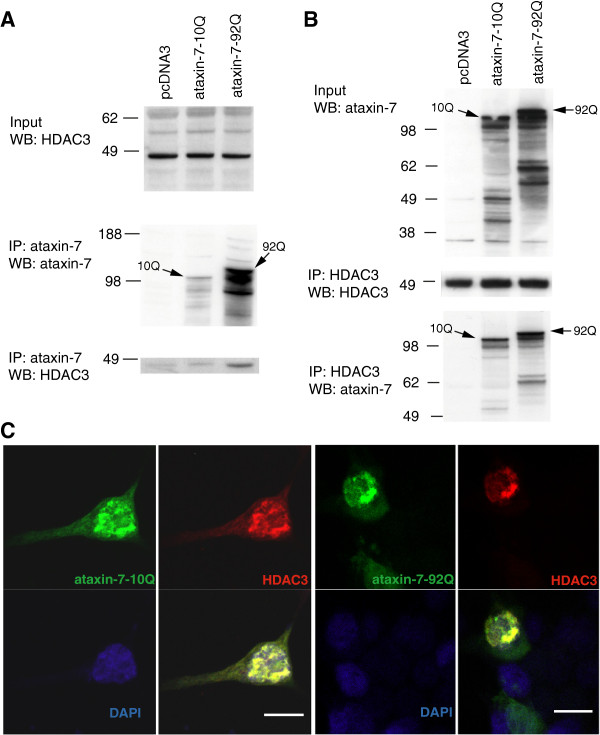
**HDAC3 and ataxin-7 physically interact and colocalize. (A)** Immunoprecipitation of HEK293T cells transiently transfected with ataxin-7-10Q (wild type) or ataxin-7-92Q (expanded), or vector. Whole cell lysates were subject to immunoprecipitation with an antibody to ataxin-7 (PA1-749), separated by SDS-PAGE and immunoblotted with an antibody to HDAC3 (N-19). **(B)** Reverse immunoprecipation with HDAC3 (B-12) and western blotting with ataxin-7 (K) supports the binding of ataxin-7 to HDAC3. **(C)** Immunocytochemistry of HEK293T cells transiently transfected with ataxin-7-10Q (wild type) or ataxin-7-92Q (expanded), and co-transfected with HDAC3. Cells were fixed and stained with antibodies to ataxin-7 conjugated to Alexa 488 (green), HDAC3 conjugated to Alexa 555 (red), and counterstained with DAPI (blue). Cells were imaged using confocal microscopy to elucidate colocalization of ataxin-7 and HDAC3. Scale bar represents 10 μm.

Based on the enhanced stability of ataxin-7 in the presence of HDAC3, we determined whether ataxin-7 and HDAC3 directly interact. Immunoprecipitation with an antibody to ataxin-7, in cells expressing HDAC3 and wild type or expanded ataxin-7, pulled down HDAC3 (Figure 2A). Interestingly, the polyQ-expanded ataxin-7 pulled down noticeably more HDAC3 than wild type ataxin-7, although there is more polyQ-expanded ataxin-7 present, as detected by ataxin-7 immunoblotting. Reverse immunoprecipitation with HDAC3 pulled down wild type and expanded ataxin-7, again at elevated levels, supporting the physical interaction of ataxin-7 and HDAC3 that may be increased in the polyQ-expanded mutant. Immunocytochemistry of co-transfected cells revealed that HDAC3 was co-localized with both wild type and expanded ataxin-7, particularly in the nucleus (Figure 2B), consistent with our co-IP results. Consistent with the physical interaction we see high-density ataxin-7 staining in the nucleus upon ataxin-7-92Q expression, indicative of the nuclear aggregates commonly detected in SCA7 models (Figure 2C).

### HDAC3 interaction modifies ataxin-7 subcellular localization and cellular toxicity

**Figure 3:**
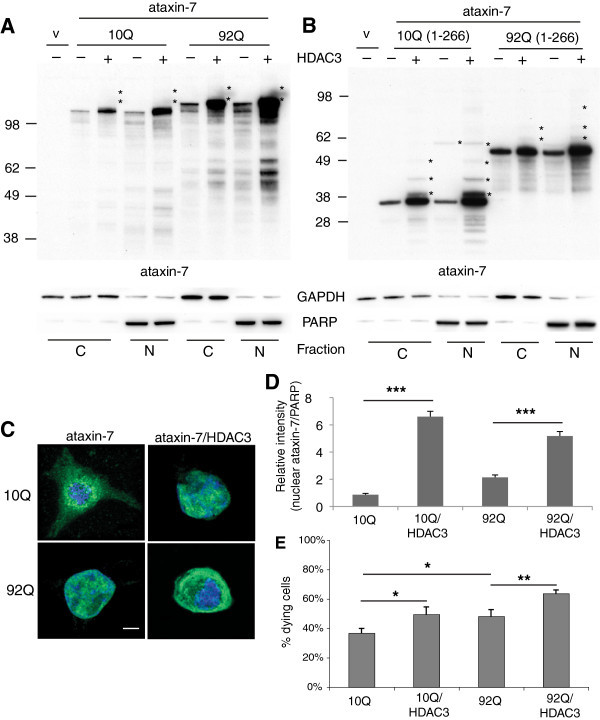
**HDAC3 alters ataxin-7 subcellular localization. (A,B)** Expression of ataxin-7 in cytoplasmic and nuclear fractions of HEK293T cells. Cells were transiently transfected with ataxin-7-10Q (wild type), ataxin-7-92Q (expanded) **(A)**, or the 1–266 N-terminal fragment of wild type and expanded ataxin-7 **(B)** and cotransfected with vector control (v) or HDAC3. Lysates were subjected to subcellular fractionation followed by western blot analysis of nuclear and cytoplasmic fractions. Ataxin-7 antibody revealed the presence of bands corresponding to the wild type, expanded and stop mutant ataxin-7. Bands of higher molecular weight of ataxin-7 are evident when ataxin-7 is co-expressed with HDAC3 (indicated by asterisks) and present in both nuclear and cytoplasmic fractions. Co-transfection of HDAC3 was confirmed by immunoblotting (data not shown). To ensure equal loading, immunoblotting with GAPDH was used for the cytoplasmic fractions and poly-ADP ribose (PARP) for the nuclear fraction. **(C)** Immunocytochemistry of transfected HEK293T cells. Cells were fixed and stained with antibodies to ataxin-7 conjugated to Alexa 488 (green), HDAC3 conjugated to Alexa 555 (red), and counterstained with DAPI (blue). Only the ataxin-7 and DAPI expression are shown, although co-transfection of HDAC3 was determined by visualization of the red track when appropriate. Cells were imaged using confocal microscopy to elucidate colocalization of ataxin-7 and DAPI. Scale bar represents 5 μm. **(D)** Quantification of ataxin-7 in nuclear fractions of HEK293T cells. Cells were transiently transfected with ataxin-7-10Q, ataxin-7-92Q and cotransfected with vector control (v) or HDAC3. Lysates were subjected to subcellular fractionation followed by western blot analysis of nuclear fractions. Quantification (n = 3) of ataxin-7 levels were normalized relative to nuclear poly (ADP-ribose) polymerase PARP (1:1000, PARP #9542, Cell Signaling Technology, Boston MA). **(E)** Cell toxicity determined by Hoescht staining. Cells were transfected with ataxin-7-10Q or expanded ataxin-7-92Q and visualized via immunocytochemistry following addition of Hoescht counterstain.

During immunocytochemical co-localization studies we noticed that ataxin-7 subcellular localization shifted upon HDAC3 overexpression and so explored this in more detail. Using biochemical subcellular fractionation, we found that HDAC3 enhanced ataxin-7 stability and posttranslational modifications in both cytoplasmic and nuclear fractions for all ataxin-7 proteins expressed (Figure 3A,B). With both the full-length and N-terminal fragments, expanded ataxin-7 is more highly expressed than wild type ataxin-7, consistent with enhanced stability of the mutant form (Figure 3C). As expected ataxin-7 was found both in the cytoplasm and nucleus of the transfected cells (Figure 3C). Interestingly, the levels of ataxin-7 protein are higher in both the cytoplasmic and nuclear fractions. Quantification of the levels of ataxin-7-10Q or ataxin-92Q in the nucleus demonstrated increased amounts of the protein in the nucleus (3–5 fold increase) (Figure 3D). This was due to increased expression of the protein in the presence of HDAC3 and not a change in the relative ratio of ataxin-7 protein in the nucleus relative to the cytoplasm.

With reports from the literature of HDAC3 enhancing neurotoxicity [27], we next evaluated whether increased expression of ataxin-7-92Q impacted cell survival. By immunocytochemistry using Hoescht staining we detected a significant increase in the number of dying cells upon HDAC3 co-expression in 293T cells (Figure 3E). Expression of expanded ataxin-7 had more dying cells (~48%) when compared to those expressing wild-type ataxin-7 (36%). Transfected cells were counted as either healthy, well-rounded nuclei or dying compact nuclei. Bars represent the average percentage of dying cells from four fields with n = 100 cells/field. Asterisks represent significant change between the ataxin-7 transfected cells and those co-transfected with HDAC3. *:p < 0.05; **:p < 0.01; ***:p < 0.005.

### Role of deacetylase activity in HDAC3 effects on ataxin-7

**Figure 4:**
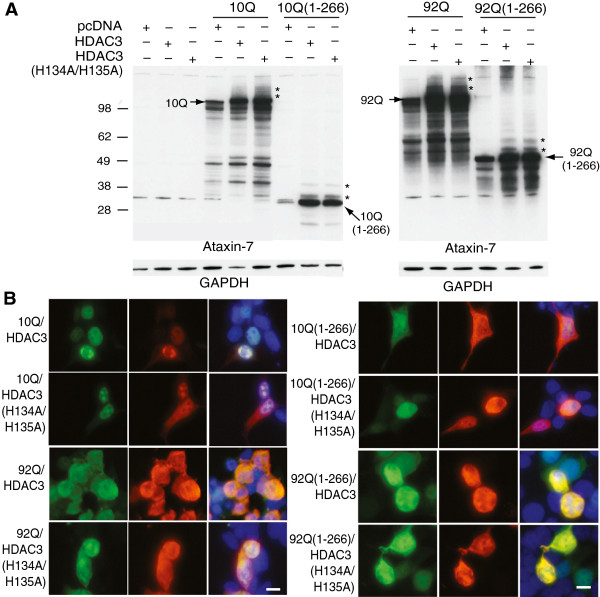
**Role of HDAC3 deacetylase activity in ataxin-7 post-translational modification and subcellular localization. (A)** Ataxin-7-10Q, ataxin-7-10Q (1–266), ataxin-7-92Q or ataxin-7-92Q (1–266) were cotransfected with vector control or HDAC3 or a catalytically-inactive mutant of HDAC3, HDAC3 (H134A/H135A), in HEK293T cells. Western blot analysis of cellular lysates with ataxin-7 (PA1-749) antibody revealed the presence of bands corresponding to the wild type, expanded and stop mutant ataxin-7. Bands of higher molecular weight that are detected when ataxin-7 is co-expressed with HDAC3 (indicated by asterisks) are also present when ataxin-7 is co-expressed with the catalytically-inactive HDAC3 mutant. Co-expression of HDAC3 was confirmed by immunoblotting (HDAC3 antibody sc17795; data not shown) and GAPDH was used as a loading control. **(B)** Immunocytochemistry of HEK293T cells transiently transfected with ataxin-7-10Q (wild type) or ataxin-7-92Q (expanded), and co-transfected with HDAC3 or the catalytically-inactive mutant HDAC3 (H134A/H135A). Cells were fixed and stained with antibodies to ataxin-7 conjugated to Alexa 488 (green), HDAC3 conjugated to Alexa 555 (red), and counterstained with DAPI (blue). Cells were imaged using confocal microscopy to elucidate colocalization of ataxin-7 and HDAC3, which can be detected by yellow co-staining. Scale bar represents 5 μm wild type.

In order to determine whether the effect of HDAC3 on ataxin-7 stability, PTMs or localization depended on its deacetylase activity we used a catalytically-inactive HDAC3 mutant. Co-expression of this form of the HDAC3 (H134A/H135A) had the same effect on the stability of ataxin-7 protein as wild type HDAC3 (Figure 4A). The presence of ataxin-7 high molecular weight bands immunoreactive to ataxin-7 was similar in the presence of either form of HDAC3 (Figure 4A). This was true for wild type and expanded ataxin-7 as well as the N-terminal fragments. Similarly, no effect was observed on the subcellular localization of ataxin-7 as detected by immunocytochemistry (Figure 4B).

### HDAC3 in mouse brain

**Figure 5:**
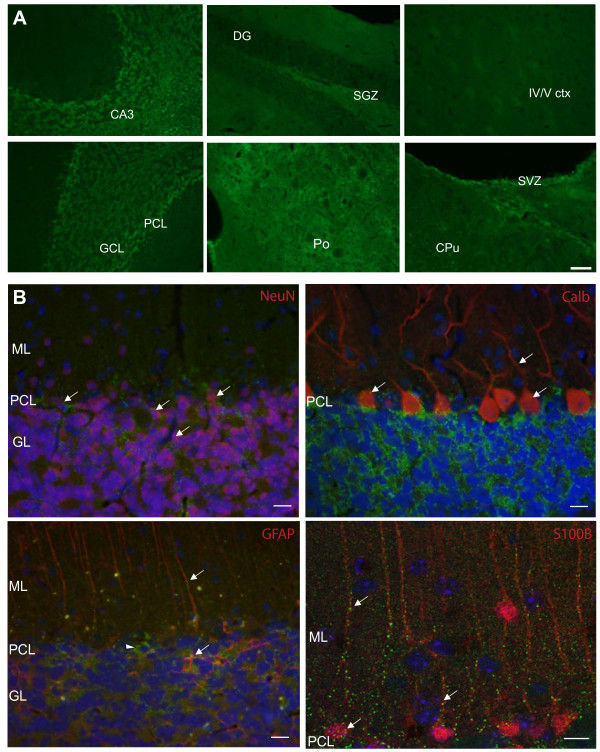
**HDAC3 expression in normal brain and cell-specific expression in the cerebellum. (A)** Immunohistochemical staining of sections from 10.5-month-old non-transgenic control mouse. Fixed tissue sections were probed with an antibody to HDAC3 conjugated to Alexa 488. Multiple mouse brain regions were captured. Top panel L-R: hippocampus CA3 region; dentate gyrus (DG) and subgranular zone (SGZ); and level IV-V motor cortex with pyramidal neurons visible; Bottom panel L-R: granular cell layer (GCL) and purkinje cell layer (PCL) of the cerebellum; pons (Po); caudate putamen (CPu) and subventricular zone (SVZ). Scale bar is 40 μM. **(B)** Immunohistochemical staining of sections from the cerebellum of 12-month-old non-transgenic control mouse. Fixed tissue sections were probed with an antibody to HDAC3 conjugated to Alexa 488 (green) and antibodies to various cell-specific markers conjugated to Alexa 555 (red) and counterstained with DAPI (blue). NeuN marks neuronal nuclei and colabeling with HDAC3 is particularly evident in the upper nuclear layer (arrows), also called the purkinje cell layer (PCL). Calbindin marks purkinje cells with HDAC3 colocalization detected both in the cell bodies and processes (arrows). GFAP marks neuroglia. Colocalization with HDAC3 is detected in the processes of Bergman glia (radial glial cells; arrows) in the molecular layer (ML) as well as in astrocytes in the granular cell layer (GCL; arrowhead). High-resolution image with S100 calcium-binding protein beta (S100β) staining specifically of Bergmann glia cells, with discrete HDAC3 colocalization in cell bodies and along processes (arrows). Scale bar represents 10 μm.

Given our findings of HDAC3 interaction with ataxin-7 and regulation *in vitro*, we next looked at HDAC3 expression *in vivo*. In order to determine if HDAC3 was expressed in regions non-transgenic of interest in SCA7 mice, we first defined HDAC3 regional and cellular localization in nontransgenic mouse brain. Immunohistochemical analysis of HDAC3 levels in the mouse brain showed that HDAC3 is expressed in discrete regions with high representation in regions relevant to SCA7 (Figure 5A). In wild type mice we detected highest expression of HDAC3 in the granular and purkinje cell layers of the cerebellum (Figure 5A, lower left panel). There was diffuse yet marked expression of HDAC3 in the pons and distinct expression of HDAC3 in the hippocampal granular layer (CA3 region), as well as subgranular and subventricular zones. HDAC3 was not highly expressed in the cortex (Figure 5A, upper right panel).

Given the high expression of HDAC3 in the cerebellum, a region of interest in SCA7, we determined which cells were expressing HDAC3 by co-labeling with cell-specific markers (Figure 5B). We found punctate expression of HDAC3 in neurons of the granular cell layer and purkinje cell layer. HDAC3 expression was detected both in the cell bodies and processes of purkinje neurons. Interestingly, HDAC3 had distinct colocalization with glia, with high-resolution imaging detecting HDAC3 particularly in the cell bodies and processes of Bergmann glia, a cerebellum-specific radial glial cell.

### HDAC3 expression and enzymatic activity in a SCA7 transgenic mouse model

Distinct colocalization of HDAC3 to cerebellar purkinje neurons and Bergmann glia, both implicated in mouse models of SCA7, lead us to explore HDAC3 expression and also HDAC activity in SCA7 transgenic mice. For this we used an established transgenic disease model, with ataxin-7 wild type (SCA7-10Q) or expanded (SCA7-92Q) expressed under the mouse prion promoter [14,16]. We used two tissue types most commonly associated with SCA7 pathology: the cerebellum and retina.

**Figure 6:**
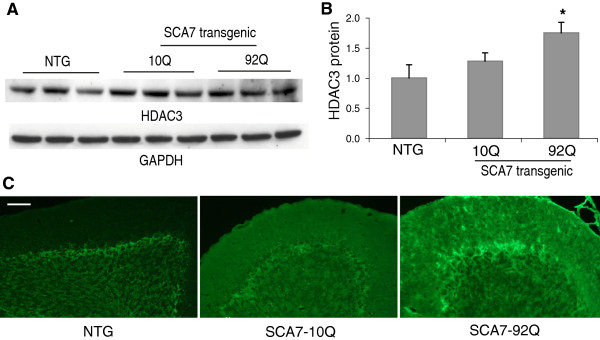
**HDAC3 expression in SCA7 tissue. (A)** Protein was extracted from cerebellar tissue of 10.5-month-old MoPrP-flag-92Q-myc transgenic mice and non-transgenic littermates and subjected to SDS-PAGE and Western blotting with an antibody to HDAC3. GAPDH was used as a loading control. **(B)** Protein bands were measured by densitometry and the expression from each genotype is denoted as an average of their HDAC3 density values (n = 3) normalized to GAPDH and shown as a ratio to non-transgenic values. Bars represent standard deviation from the mean and asterisks is significance at p < 0.05. **(C)** Immunohistochemical staining of 9-month-old MoPrP-flag-10Q-myc and MoPrP-flag-92Q-myc transgenic mice and non-transgenic littermate controls. Fixed cerebellar tissue sections were probed with an antibody to HDAC3 conjugated to Alexa 488. Scale bar represents 50 μm.

In the cerebellum, we found that HDAC3 protein levels were significantly elevated in 9-month-old SCA7-92Q transgenic mice by western blot analysis (Figure 6A,B). The increase in expression of HDAC3 is likely much higher as we found a significant portion of the protein was retained in the loading well for the SCA7-92Q lysates most likely due to aggregation (data not shown). This increase was strikingly evident in immunohistochemical analysis with a substantial increase in HDAC3 expression in SCA7-10Q and SCA7-92Q transgenic mice (Figure 6C). In wild type animals HDAC3 expression is confined to the granular cell layer, whereas in transgenic animals HDAC3 is expressed in cells in the molecular layer. In SCA7-92Q mice there is high expression of HDAC3 in the molecular layer, with glial cell projections, in the granular cell layer and also in the soma and projections of the remaining purkinje cell neurons, despite obvious degeneration of this cell type consistent with disease progression. In order to determine whether these protein changes lead to discernible effects on enzymatic activity we examined global acetylation levels.

**Figure 7:**
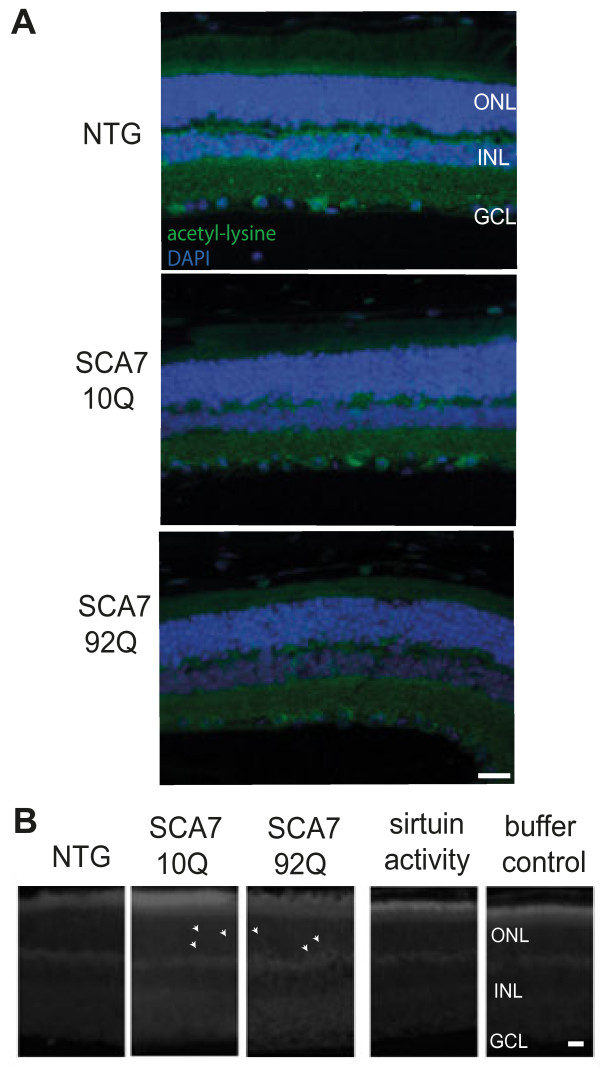
**Acetylation in SCA7 transgenic mice. (A)** Immunohistochemical staining of 10.5-month-old SCA7 transgenic mice and nontransgenic littermate controls. Fixed retinal tissue sections were probed with an antibody to acetyl-lysine conjugated to Alexa 488 (green) and counterstained with DAPI (blue). Scale bar represents 20 μm. **(B)** HDAC activity in the retina *in vivo*. HDAC activity was detected *in situ* using a deacetylase substrate, particularly in the pigment epithelium and segment layers of the retina. Activity due to sirtuins (HDAC III class) was similar to buffer control (panels shown represent activity in SCA7-10Q tissue) as detected by HDAC I/II class specific inhibition with TSA (100 μM). Arrowheads indicate fluorescent cells. ONL: outer nuclear layer; INL: inner nuclear layer; GCL: ganglion cell layer. Scale bar represents 10 μm.

In the retina of wild type mice, immunoreactivity of acetyl-lysine was detected particularly in ganglion cells and the mixed population of cells in the inner nuclear layer, with some labeled cells detected in the outer nuclear layer (photoreceptors) (Figure 7A). Acetyl-lysine intensity is decreased in SCA7 transgenic animals, particularly in the inner and outer nuclear layers. Note that there is a marked decrease in the number of cells in the outer and inner nuclear layers in the SCA7-92Q transgenic retina, consistent with neurodegeneration of photoreceptor cells and bipolar/ganglion cells respectively.

Since the level of acetylated proteins is decreased in the SCA7 mice, we measured deacetylase activity *in situ*. Deacetylase activity in the retina of SCA7 transgenic mice was increased, particularly in the segment layers of the retina containing photoreceptor rods and cones and also in distinctive cells in the outer nuclear layer containing photoreceptor cell nuclei (marked by arrowheads) (Figure 7B). The contribution of sirtuins to this activity was minimal, as assessed using an HDAC I/II specific inhibitor. Tissue degeneration is marked in the SCA7-92Q retinal layers, and this could contribute to a seemingly more uneven increase in deacetylase levels.

## Discussion

Many polyglutamine diseases, including SCA7, feature transcriptional deregulation associated with altered acetylation state [17,34,35] and cellular toxicity that is reversible by HDAC inhibition [35]. We have previously shown that acetylation and deacetylation affect ataxin-7 stability [16]. As ataxin-7 functions in a transcriptional repressor complex, one proposed mechanism for deregulation is aberrant interaction of the polyQ-expanded protein with factors that regulate acetylation/deacetylation. In this study we focused on a role for HDAC family members in ataxin-7 regulation. This is a critical study given that HDAC inhibitors have been found to ameliorate symptoms in many models of trinucleotide repeat diseases, including Huntington’s disease (HD) and Freidrich’s ataxia [31,32]. Our study indicates that HDAC inhibitors may also be a target for SCA7 treatment.

In this study we identified HDAC3 as a protein binding-partner of ataxin-7. We showed that HDAC3 modifies ataxin-7 stability, PTM and subcellular localization. Enhanced stability of ataxin-7 could be due to increased production or to reduced degradation. Our previous study supports the latter conclusion, as we have previously found that modification of ataxin-7 at lysine-257, by SUMOylation or acetylation, enhances ataxin-7 stability [16]. Consistent with this study, the PTM-deficient mutant K257R does not have increased protein levels or modifications with HDAC3 co-expression. Future studies may uncover the role of modifications at lysine-257 on HDAC3 effects. Interestingly, we did not find a differential effect on ataxin-7 stability or PTMs using a catalytically-inactive mutant of HDAC3 compared to the wild type HDAC3. This indicates that the protein-protein interaction between ataxin-7 and HDAC3, not its deacetylase activity, may be involved in enhancing ataxin-7 levels and modifications. An alternate explanation is that another HDAC family member that is in complex with HDAC3 is responsible for modifying ataxin-7.

A major effect of the interaction between ataxin-7 and HDAC3 is increased levels of the mutant ataxin-7 protein, with enhanced nuclear expression in cells overexpressing HDAC3, concomitant with increased cell toxicity. Subcellular redistribution may be due to ataxin-7 shuttling with HDAC3, which is known to undergo nuclear/cytoplasmic shuttling as part of a transcriptional corepressor complex [24]. While this effect on localization may occur via PTM regulation, previous studies have shown that SUMOylation does not effect subcellular localization of ataxin-7 [33] and our study further indicates that it is not a result of HDAC enzymatic activity.

As with many neurodegenerative disease proteins, ataxin-7 is ubiquitously expressed in the brain and yet the disease manifests in specific brain regions. One proposition is that regional or cell-specific expression of interacting proteins, such as HDAC family members, contributes to selectivity of disease progression [36,37]. Given the varied effects of HDAC3 interaction on ataxin-7 *in vitro*, we explored the region-specificity of HDAC3 in the mouse brain. One previous study analyzed HDAC3 mRNA in the brain, with high expression noted in granular cells of the cerebellum and hippocampus [25]. Our study in normal mouse brain found HDAC3 protein highly expressed in the cerebellum, with moderate expression in pons and hippocampus, in particular the subgranular zone and also caudate putamen, particularly the subventricular zone. HDAC3 knockout mice are embryonic lethal due to cardiomyopathy [26]. However, conditional knockout mice have indicated a role for HDAC3 in brain function as specific deletion in the hippocampus implicated HDAC3 as a critical negative regulator of long-term memory formation [38], consistent with our findings of marked HDAC3 expression in the hippocampus. Our expression analysis suggests HDAC3 may also play a role in neurogenesis, which is consistent with previous studies showing that HDAC3 is expressed in developing rat brain and that HDAC activity is crucial for determining cell progenitor fate [39].

Given the high expression of HDAC3 in the cerebellum, and the major degeneration of this region in SCA7, we determined the cell-specific expression of HDAC3. Neuronal and glial-cell markers showed HDAC3 to be highly expressed in the Purkinje cell layer, with distinct colocalization with Bergmann glial cell soma and processes. SCA7 animal models have revealed that polyQ-expanded ataxin-7 dysfunction in either neurons or glia of the cerebellum leads to behavioural phenotypes and neurodegeneration, but that rescue of ataxin-7 function in both cell types is required to reverse the SCA7 phenotype [40,41]. This non-cell autonomous feature indicates that SCA7 degeneration resulting from ataxin-7 dysfunction results from interactions in purkinje cells and Bergmann glia, both of which our study shows express HDAC3.

Given this remarkable expression pattern we next assessed HDAC3 expression in a well-characterized SCA7 transgenic mouse model. In the cerebellum we found increased HDAC3 protein levels in the SCA7 polyQ-expanded transgenic mice. In cells we showed that HDAC3 and ataxin-7 interaction was enhanced with polyQ-expanded ataxin-7, which we hypothesize could lead to increased stability of the HDAC3 protein in SCA7. Interestingly, increased expression of HDAC3 protein has recently been shown to be highly toxic to cerebellar and cortical neurons but not to non-neuronal cells [27], which may explain why neurons selectively die despite our evidence for high expression of HDAC3 in glia.

In the retina we found decreased acetyl-lysine levels in photoreceptor cell body layers, concomitant with increased deacetylase activity. These changes are consistent with previous studies that evaluated promoter occupancy of the SCA7-92Q mice relevant to histone H3 acetylation and found a significant decrease with disease progression at 6 and 11 weeks of age [8]. Further, in HD mouse models histone H3 acetylation is decreased during disease [42]. However, our study is particularly interesting as it is known that HDAC activity requires binding to cofactors [22] and previous studies have show that HDAC3 interaction with a transcriptional corepressor, N-CoR, increased HDAC activity [21]. HDAC3 interaction with ataxin-7, a constituent component of transcriptional corepressor complexes, could also potentially increase HDAC activity, enhancing toxicity.

From our findings we hypothesize that HDAC3 interaction with polyQ-expanded ataxin-7, particularly in the pathological regions in SCA7, increases HDAC3 stability and HDAC activity, leading to transcriptional deregulation, toxicity and neurodegeneration. Future studies would directly analyze this role by looking at the affect of altered HDAC3 levels in a SCA7 transgenic model, particularly on behavioural phenotypes, cerebellar degeneration and morbidity. A pathogenic role for HDAC3 in SCA7 must be further assessed in transgenic mouse models before any conclusions are drawn, although it is consistent with previous studies that HDAC inhibitors with high affinity to HDAC3 complexes [43] may ameliorate deficits in polyQ disease models. In a mouse model of Freidich’s Ataxia, treatment with HDAC inhibitors with an HDAC3 preference enhanced motor coordination and ameliorated multiple pathological deficits [32]. Using a similar compound another study found that HDAC inhibition reduced behavioural phenotypes and pathology as well as partially correcting transcriptional deregulation in a mouse model of HD [31] although other studies have questioned the specificity of the drug or did not reproduce all the beneficial effects found in the first study [44,45]. Furthermore, HDAC3 knockdown in a *C. elegans* HD model suppressed neurotoxicity [30], consistent with our findings of HDAC3 enhancing cellular toxicity. In contrast to these latter reports, one study found no benefit of partial HDAC3 knockdown in a genetic cross with an HD mouse model [46]. However, recent studies suggest that the wild-type Htt binds to HDAC3 and dissociation of HDAC3 when mutant Htt is present is essential for toxicity [47]. We propose that the physical interaction of HDAC3 with ataxin-7, and other expansion disease proteins resulting in cellular toxicity (perhaps with distinct mechanisms), makes HDAC3 a target for intervention in these diseases. In support of a role for HDAC3 in trinucelotide repeat diseases, a recent report found that HDAC3 promotes instability, leading to expansions of trinucleotide repeats in cell and animal models [48].

In summary, we found that HDAC3 and ataxin-7 physically interact, leading to increased stability, enhancing post-translational modifications and increased subcellular localization of ataxin-7. Furthermore, we detected altered HDAC3 levels, acetyl-lysine levels and deacetylase activity in a transgenic mouse model of SCA7 and found HDAC3 to be highly expressed in both neurons and glia in the cerebellum of nontransgenic and SCA7 transgenic mice. Further studies will elucidate the physical nature of the interaction between ataxin-7 and HDAC3, the effect of polyQ-expansion on the interaction and levels of HDAC3 and on SCA7 pathogenesis, and whether alteration of HDAC3 levels may be a viable pharmacological target. With reports of HDAC3 binding to three different ataxin proteins [18,19,47], and HDAC3-specific inhibition ameliorating disease in animal models [31,32,34], our study adds to growing evidence for HDAC3 modulation as a target for multiple neurodegenerative diseases.

## Methods

### SCA7 transgenic mice

Production and characterization of SCA7 transgenic mice, with ataxin-7 containing 10 or 92 CAG repeats expressed under the direction of the prion promoter (PrP) has been described previously [14]. Expression levels for PrP-mycFlag-SCA7-10Q-Line A305 and PrP-mycFlag-SCA7-92Q-Line B306 were determined in the initial characterization [16]. Transgenic mice in the PrP-mycFlag-SCA7-92Q-Line 306 overexpress ataxin-7 two-fold relative to endogenous levels, have retinal pathology by 12-weeks of age and have a shortened lifespan (9 months). At the Buck Institute for Research on Aging, an AAALAC international B306 accredited institution (Unit #001070), all procedures were approved by the Institutional Animal and Use Committee (A4213-01).

### Plasmid constructs

Ataxin-7 cDNA was cloned into pcDNA3.1 (Invitrogen, Carlsbad, CA), with inserts containing either 10 or 92 CAG repeats, and a stop mutant created by site-directed mutagenesis at amino acid position 266, as previously published [16]. Site-directed mutagenesis was performed to generate a K257R amino acid substitution as previously described [16]. All constructs were sequenced to confirm that the appropriate mutation was introduced and CAG repeat length was not altered. For co-expression studies, plasmids encoding HDACs 2–8 were purchased from Origene (Rockville, MD): HDAC2 (#SC110918), HDAC3 (#SC112704), HDAC6 (#SC111132), HDAC8 (#SC321829) or Addgene (Cambridge, MA): HDAC4 (#13821), HDAC5 (#13822). HDAC7 plasmid was a gift from Dr. Hung-Yin Kao and the HDAC3 catalytically-inactive mutant construct (H134A/H135A substitutions) was a gift from Dr. Eric Verdin.

### Cell culture, transfection and harvesting

HEK293T cells were cultured in DMEM (Mediatech, Manassas, VA) containing 1% penicillin/streptomycin (25 units/mL each) and 10% heat-inactivated fetal bovine serum (DMEM complete) unless otherwise specified. Transient transfections were performed using lipofectamine 2000 (L2000, Invitrogen) according to manufacturer’s instructions. For total protein extractions, cells were seeded in 6-well dishes at approximately 5 × 10^5^ cells/well. Each well was transfected with 4 μg total DNA by L2000 in DMEM lacking serum and antibiotics. Following 72 h incubation at 37°C, cells were harvested, lysed in Mammalian Protein Extraction Reagent (MPER; Thermo Scientific, Rockford, IL) containing protease inhibitors (Complete Mini, Roche Applied Science, Mannheim, Germany), sonicated and spun at 12,000 g for 15 min to remove undigested cellular components. Protein concentration was determined using a BCA assay (Pierce, Rockford, IL). For subcellular fractionation assays, 6 × 10^6^ cells were seeded in 10 cm plates, transfected with 25 μg DNA by L2000 in serum-free DMEM and incubated for 72 h at 37°C. For immunocytochemistry, including cellular toxicity assays, cells were seeded in 8-well slides at 6–10 × 104 cells/well. Each well was transfected with 1.2 μg total DNA in serum-free DMEM and processed following 48 h incubation at 37°C.

### Subcellular fractionation

For subcellular fractionation protein assays, transfected HEK293T cells were harvested and nuclear and cytoplasmic fractions were separated using the NE-PER Nuclear and Cytoplasmic Extraction Kit (Thermo Fisher Scientific, Rockford Illinois) according to the manufacturer’s instructions. Briefly, cells were harvested in trypsin-EDTA (Mediatech) and approximately 6 × 10^6^ cells were spun at 500 g for 3 min. Supernatant was removed and approximately 50 μL of cells recovered. Cytoplasmic Extraction Reagents (CERI and CERII) were added to these cells, which were then vortexed at high-speed and spun at 16,000 g for 5 min at 4°C. The cytoplasmic protein fraction was recovered from the supernatant fraction. The pellet was resuspended in Nuclear Extraction buffer (NER) by intermittent high-speed vortexing over 40 min and the sample was centrifuged at 16,000 g for 10 min at 4°C. The nuclear protein was separated into the supernatant fraction. Protein concentrations of the nuclear and cytoplasmic fractions were determined using a BCA assay and 10 μg of protein were subjected to western blotting.

### Immunoprecipitation

Pull-down assays of transfected HEK293T cells were undertaken using Protein G Sepharose beads (GE Healthcare, Uppsla, Sweden), washed and used according to the manufacturer’s instructions. Lysates (500 μg) were pre-cleared by incubation with beads without the antibody for 1 h at 4°C with rotation. Pre-cleared lysates were incubated with primary antibody, 3 μL ataxin-7 (PA1-749) or 5 μL HDAC3 (sc-17795), overnight with rotation at 4°C. Antibody-incubated lysates were transferred to the bead slurry and immunoprecipitated for 2 h at 4°C with rotation. Immunodepleted sample was removed after centrifugation at high-speed for 3 min. Bound beads were then washed rigorgously in lysis buffer (MPER with proteinase inhibitors, as described above) and once in HEPES made up in lysis buffer without proteinase inhibitors to remove salts. Beads were precipitated by boiling in water with loading buffer and DTT (10 mM) for 10 min and spun briefly to collect beads. Half of the precipitate was used for western blotting, along with immunodepleted samples and 10 μg of input protein.

### Western blotting of transfected cells

For western blotting of transfected HEK293T cells, 10 μg of protein containing DTT (10 mM) was heated at 95°C for 10 min. Samples were subjected to SDS-PAGE using 4-12% NuPage Bis-Tris (Invitrogen) gels under reducing conditions in MES SDS running buffer (Invitrogen). Gels were run at 200 V for 70 min, transferred to 0.45 μM nitrocellulose membrane (Whatman, Dassel, Germany) in NuPage transfer buffer (Invitrogen) with 10% methanol and western transfer undertaken at a constant 350 mA for 1 h. Membranes were blocked in TBS with 0.1% Tween 20 (TBS-T) containing 5% milk for 1 h. Primary antibodies were diluted in blocking solution at 1:1000 for ataxin-7 (PA1-749, Thermo Scientific, Rockford, IL), 1:100 for HDAC3 (sc-17795, Santa Cruz Biotechnology), 1:5000 for GAPDH (MAB734; Chemicon, Temecula, CA), 1:1000 for β-actin (#4967S; Millipore, Billerica, MA) and 1:500 for poly-ADP ribose (PARP; SA-253, Enzo Life Science, Farmingdale, NY). Immunoblots were developed with a peroxidase-conjugated secondary antibody and enhanced chemiluminescence.

### Immunocytochemistry

Cells were fixed by adding 8% paraformaldehyde directly to culture medium to a final concentration of 4%. Cells were incubated for 20 min with gentle agitation then washed with PBS at room temperature (RT). Cells were permeabilized in 0.25% Triton in TBS for 15 min, washed in TBS, and blocked in 10% NDS in TBS for 1 h. Primary antibody was incubated with cells overnight at 4°C after dilution in TBS containing 1% BSA at 1:200 for ataxin-7 (K antibody: [14,16] and 1:20 for HDAC3 (sc-17795, Santa Cruz Biotechnology). Cells were then washed in TBS and secondary antibody in TBS containing 1% BSA was applied for 1 h at RT. Ataxin-7 was labeled with 1:500 Alexa 488 donkey anti-rabbit and HDAC3 was labeled with 1:500 Alexa 555 donkey anti-mouse. For the cell toxicity assay, 1:5000 Hoescht 33342 (10 mg/ml; H21492, Invitrogen) was included in the secondary antibody incubation. A no primary antibody control was included in each experiment to detect background fluorescence. Cells were washed, dried and mounted with ProlongGold +/− DAPI (Invitrogen). Slides were cured overnight. Confocal images were obtained using a Zeiss LSM 510 NLO microscope and Zeiss LSM Image Browser software. Laser settings used were 488 Ar to capture ataxin-7, 543 He/Ne for HDAC3 and Chameleon Ultra tuned to 780 nm for DAPI acquisition. Following capture, all images were compiled in Adobe Photoshop using identical parameters for each channel. For the cellular toxicity assays, 100 cells/field were counted in two separate fields for each transfection condition and this was duplicated in a separate transfection.

### Protein extraction and western blotting of SCA7 transgenic mouse tissue

Protein was extracted from the cerebellum of 9-month old mice: PrP-mycFlag-SCA7-10Q-Line A305, PrP-mycFlag-SCA7-92Q-Line B306 and non-transgenic littermate controls. Cerebella were homogenized in 10 ml/g tissue of Tissue Protein Extraction Reagent (TPER; Thermo Scientific) containing protease inhibitors, DNase (10 U/mL), MgCl2 (1.2 mM), epoxymycin (1 μM) phosphatase inhibitors (Phosphatase Inhibitor Cocktail Set II, Calbiochem, La Jolla, CA) and HDAC inhibitors (50 μM TSA, 30 μM sodium butyrate, 30 mM nicotinamide). Tissue was homogenized, snap frozen at −80°C overnight, defrosted at RT to enhance cell lysis, sonicated and spun at 12,000 g for 15 min. Supernatant and pellets were kept for analysis. Protein concentration was determined using a BCA assay (Pierce, Rockford, IL) and 40 μg of proteins were run on polyacrylamide gels in MES buffer for 70 min at 200 V. Proteins were transferred to a 0.45 μm nitrocellulose (Whatman, Dassel, Germany) membrane at 20 V overnight (14 h) at 4°C in NuPAGE tranfer buffer with 10% methanol. Band intensity was quantified using NIH ImageQuant TL v2005. Membranes were blocked in TBS with 0.1% Tween 20 (TBS-T) containing 5% milk for 1 h. Primary antibodies were diluted in blocking solution at 1:1000 for ataxin-7 (PA1-749; Affinity Bioreagents), 1:2500 for HDAC3 (ab7030, Abcam, Cambridge, MA) and 1:1000 tubulin (T4026; Sigma-Aldrich, St Louis, MI) Immunoblots were developed with a peroxidase-conjugated secondary antibody and enhanced chemiluminescence (Pierce).

### Immunohistochemistry of SCA7 transgenic mouse brain sections

SCA7 mice were perfused with PBS and then 4% PFA in PBS. The eyes and brain were removed and embedded in paraffin blocks. Brain and retinal tissue was cut into 7 μM sections on a microtome. Paraffin-embedded sections were washed with xylene twice for 5 min to deparaffinize the tissue. Sections were re-hydrated in consecutive ethanol washes (100% to 70%) before resuspension in 1X Tris-buffered saline for 15 min. All washes were performed at RT. For antigen retrieval, sections were microwaved in 10 mM citrate buffer, pH 6.0, for 5 min at 40% power in a 1100 W microwave oven (Sanyo). Sections were allowed to cool in the same buffer for 20 min at RT and were then transferred to TBS for 10 min. For immunostaining, sections were blocked in 10% normal donkey serum in TBS for 1 h at RT. Ataxin-7 (1:200; K antibody), HDAC3 (1:20 for retinal tissue and 1:500 for cerebellar tissue; Abcam ab7030), acetyl-lysine (1:250; Abcam ab21623), glial fibrillary acidic protein (GFAP; 1:500; Sigma G3893), neuronal nuclear marker (NeuN; 1:200; Millipore MAB377), calbindin (1:500; C9848 Sigma) and S100 calcium binding protein beta (S100β; 1:1000; Abcam ab11178) primary antibodies were diluted in 1% BSA in TBS and incubated overnight at 4°C. All subsequent steps were performed at RT. Sections were washed in TBS. Alexa 488 donkey anti-rabbit secondary antibody (1:500 in 1% BSA in TBS) was applied to sections for 1 h. Following incubation in secondary antibody, sections were washed in TBS and mounted in ProlongGold with DAPI. Slides were cured overnight. Epifluorescence images were captured on a Nikon Eclipse E800 microscope. Confocal images were obtained using a Zeiss LSM 510 NLO microscope and Zeiss LSM Image Browser software.

### HDAC activity assay

*In situ* HDAC activity was determined in retinal sections using the Fluor-de- Lys™ HDAC flurometric activity assay (Enzo Life Sciences International Inc, Plymouth Meeting, PA) previously modified for use in tissue [49]. Briefly, paraformaldehyde-fixed retinal sections (7 μM) were incubated with deacetylase substrate (200 μM) in assay buffer (50 mM Tris/Cl, pH 8.0, 137 mM NaCl, 2.7 mM KCl, 1 mM MgCl2) for 3 h at RT. HDAC I/II -specific inhibitor, trichostatin A (100 μM; Sigma, Steinham, Germany), was added to some sections to determine the influence of sirtuin activity. Buffer-only controls were included in all experiments. After incubation, sections were washed briefly in PBS and then fixed in methanol at −80°C for 20 min. Freshly prepared Fluor-de- Lys™ developer (0.5X) in assay buffer was added and sections were coverslipped and immediately visualized on a Nikon Eclipse E800 microscope in the DAPI range (excitation 350–380 nm; emission 440–460 nm).

## Abbreviations

SCA7: Spinocerebellar ataxia type 7; polyQ: Polyglutamine; STAGA: Spt/Ada/Gcn5 acetylase; HDAC: Histone deacetylase; HAT: Histone acetyltransferase; TFTC: TATA-BP free TAF-containing complex; PTM: Posttranslational modification.

## Competing interests

The authors declare that they have no competing interests.

## Authors’ contributions

CED carried out the experiments, participated in the study design, performed statistical analysis and drafted the manuscript. MCA, CR, TP and CV carried out experiments and performed statistical analysis. LME conceived the study and participated in its design and coordination and helped to draft the manuscript. All authors read and approved the manuscript.
